# The Novel Relationship between Urban Air Pollution and Epilepsy: A Time Series Study

**DOI:** 10.1371/journal.pone.0161992

**Published:** 2016-08-29

**Authors:** Chen Xu, Yan-Ni Fan, Hai-Dong Kan, Ren-Jie Chen, Jiang-Hong Liu, Ya-Fei Li, Yao Zhang, Ai-Ling Ji, Tong-Jian Cai

**Affiliations:** 1 Department of Epidemiology, College of Preventive Medicine, Third Military Medical University, Chongqing, China; 2 Information Department Medical Record Room, Second Affiliated Hospital, Fourth Military Medical University, Xi’an, China; 3 Department of Environmental Health, School of Public Health, Fudan University, Shanghai, China; 4 School of Nursing, University of Pennsylvania, Philadelphia, Pennsylvania, United States of America; 5 School of Public Health, Fourth Military Medical University, Xi'an, China; University of Catanzaro, ITALY

## Abstract

**Background and purpose:**

The data concerning the association between environmental pollution and epilepsy attacks are limited. The aim of this study was to explore the association between acute air pollution exposure and epilepsy attack.

**Methods:**

A hospital record-based study was carried out in Xi’an, a heavily-polluted metropolis in China. Daily baseline data were obtained. Time-series Poisson regression models were applied to analyze the association between air pollution and epilepsy.

**Results:**

A 10 μg/m^3^ increase of NO_2_, SO_2_, and O_3_ concentrations corresponded to 3.17% (95%Cl: 1.41%, 4.93%), 3.55% (95%Cl: 1.93%, 5.18%), and -0.84% (95%Cl: -1.58%, 0.09%) increase in outpatient-visits for epilepsy on the concurrent days, which were significantly influenced by sex and age. The effects of NO_2_ and SO_2_ would be stronger when adjusted for PM_2.5_. As for O_3_, a -1.14% (95%Cl: -1.90%, -0.39%) decrease was evidenced when adjusted for NO_2_. The lag models showed that the most significant effects were evidenced on concurrent days.

**Conclusions:**

We discovered previously undocumented relationships between short-term air pollution exposure and epilepsy: while NO_2_ and SO_2_ were positively associated with outpatient-visits of epilepsy, O_3_ might be associated with reduced risk.

## Introduction

As one of the most prevalent neurological diseases, epilepsy affects more than 50 million people worldwide with a higher prevalence rate in low-income countries [1]. A meta-analysis reports that the median prevalence of epilepsy was 5.8‰ in developing countries [2]. However, the etiology and the mechanisms involved are not clear.

Air pollution exerts great threats to human health, especially diseases of respiratory and cardiovascular systems [3,4]. In recent years, the relationship between air pollution and nervous system diseases has been recognized gradually. Calderon *et al* report that the sustained exposure to air pollutants can seriously affect pediatric central nervous system [5]. More importantly, the association between air pollution and neurodegenerative diseases is widely accepted [6].

To date, limited studies have been performed regarding the influence of air pollution on epilepsy. Although improved substantially, China has the worst air pollution in the world [7]. The industrialization, urbanization, and increased vehicle use result in the increased air pollution in major cities [8]. In this study, we investigated the effects of urban ambient air pollution on the attack of epilepsy in Xi’an, a heavily-polluted metropolis in China. We hope our data can provide clues for the associations between air pollution and epilepsy.

## Methods

### Data collection

Xi’an, with an area of 10,108 km^2^ and a resident population of 8.262 million in 2014, is the largest city in northwestern China. It experiences some of the worst air pollution among China’s cities [9]. Here, we limited the area in urban districts of Xi’an, an area of 3,586 km^2^ with a resident population of 6.565 million.

Daily outpatient data were obtained from Tangdu Hospital, one of the largest hospitals in western China. In the outpatient department, the physicians enter medical record data for each patient into the computer system, including individual characteristics (such as gender, age, and residence) and diagnoses. In this study, daily numbers of epilepsy outpatient-visits for epilepsy from urban areas of Xi’an between January 1, 2013 and December 31, 2014 were included. Patient records were de-identified and daily aggregated data were calculated for analysis. There was no individual interaction with patients. The protocol was approved by the Ethics Committee of Third Military Medical University.

Daily (24h) air pollution data including particulate matter less than 10 μm in aerodynamic diameter (PM_10_), particulate matter less than 2.5 μm in aerodynamic diameter (PM_2.5_), carbon monoxide (CO), nitrogen dioxide (NO_2_), sulfur dioxide (SO_2_), and ozone (O_3_) from January 1, 2013 to December 31, 2014 were obtained from China National Environmental Monitoring Center. The daily concentrations for pollutants were averaged from the available data of thirteen fixed-site automated monitoring stations in urban areas of Xi’an. All the monitored results reflected the general urban levels according to their strategic location designs. In order to adjust for the potential confounding effects, daily weather data (mean temperature and relative humidity) were collected from China Meteorological Bureau.

### Statistical methods

Risk of epilepsy and pollutants and weather data were based on a larger population, so we assumed a Poisson distribution. A generalized additive model (GAM) was applied. The epilepsy outpatient-visits, air pollutants, and weather data were linked by date, so we used time-series model to analyze. In order to examine the effect of air pollutants on outpatient-visits for epilepsy, we controlled the potential confounders such as long-term trend, the day of the week (DOW), and meteorological factors. We fitted non-parametric smoothing terms for the trend on days and a dummy variable for DOW. We applied natural smooth (ns) functions of calendar time. Akaka’s Information Criterion (AIC) was used to determine how well the models fitted the data and smaller AIC values of per year for time trends indicated the preferred model (S1 Table). The effects were stable when 7 or 8 degrees of freedom per year for time were used. Considering the results from AIC and other studies that had been published [10], 7 degrees of freedom (df) was found to be the best suitable for our current study. We incorporated ns functions of mean temperature (6 df) and relative humidity (3 df) to adjust for the potential nonlinear confounding effects of weather conditions based on published literature [11]. On the other hand, we compared the estimated effects by different degrees of freedoms per year for time, and the stable estimated effects suggested that the basic model was suitable (S2 Table). We also considered days of week and public holidays in the models as indicator variables. We defined lag effects of different days including both single-day lag from lag 0 to lag 7 and moving average of lag 07 (concurrent day and previous 7 days). As for air pollutants, we examined the delay days on the effects. In addition to single-day lag from lag 0 to lag 7, lag 07 was used to estimate the cumulative effects of pollutants. Residuals of each model were examined to check whether there were discernible patterns and autocorrelation by means of residual plots and the partial autocorrelation function (PACF) plots (S1 Fig). In addition, we also checked the lag effects for temperature, which could reflect the stability of models (S2 Fig). The independent model is described below:

$$logE\left({\text{Y}}_{\beta }\right){\;=\;\beta \text{Z}}_{\text{t}}\text{+ DOW + ns }\left(\text{time, df}\right)\;+\text{ ns }\left(\text{temperature, 6}\right)\;+\text{ ns }\left(\text{humidity, 3}\right)\;+\text{ intercept}$$

Here *E* (Y_β_) means the expected number of outpatient visits at day t; β represents the log-relative rate of outpatient visits associated with a unit increase of pollutant concentration; Zt indicates the pollutant concentrations at day t; DOW is day of the week effect; ns(time,df) is the natural spline function of calendar time; and ns(temperature/humidity,6/3) is the natural spline function for temperature and humidity with 6/3 df.

After establishment of the basic models, we determined which pollutants had relationship with epilepsy, investigating the effects of lag days for single pollutants. Second, we investigated whether the associations between main pollutants and epilepsy were sensitive to the adjustment: other pollutants were included one by one as potential co-pollutants with major pollutants at all lag structures. Besides the above methods, we also performed sex- and age- specific analyses and plotted exposure–response relationship curves for main pollutants.

We considered *P*<0.05 to be statistically significant. All analyses were conducted by R software (version 2.15.1) using the mgcv package. Unless specified otherwise, the pollutant effect was generally expressed as a percentage increase of outpatient epilepsy visits with a 10-μg/m^3^ increase of pollutants per day (95% confidence intervals (CIs)).

## Results

Table 1 summarizes basic descriptive statistics. There were 20,368 epilepsy outpatient-visits during January 1, 2013 and December 31, 2014, including 12,041 males and 8,327 females. The average pollutant concentrations were 169 μg/m^3^ for PM_10_, 91 μg/m^3^ for PM_2.5_, 38.6 μg/m^3^ for SO_2_, 51.3 μg/m^3^ for NO_2_, 1.939 mg/m^3^ for CO, and 100 μg/m^3^ for O_3_. Meanwhile, the average daily temperature and humidity were 15.6°C and 61%.

**Table 1:** Descriptive statistics for daily morbidity, concentrations of air pollutants, and weather conditions.

	Mean	Min	Max	P25	P50	P75	SD
Epilepsy	28	1	72	15	27	38	15
Sex							
Male	16.49	0	48	9	16	22	9.32
Female	11.4	0	34	6	11	16	6.77
Age							
Children (<18)	10.35	0	33	5	9	15	6.47
Adult (18–59)	16	0	51	9	15	22	9.18
Elderly (>59)	1.547	0	8	0	1	2	1.48
Air pollutant concentrations (24-h average)							
SO_2_ (μg/m^3^)	38.6	3.3	170.0	15.0	27.0	53.0	32.3
NO_2_ (μg/m^3^)	51.3	14.7	141.0	37.2	47.9	62.0	19.4
CO (mg/m^3^)	1.939	0.747	6.219	1.289	1.685	2.325	0.92
O_3_ (μg/m^3^)	100	7	311	50	87	144	63
PM_2.5_ (μg/m^3^)	91	10	606	44	66	105	79
PM_10_ (μg/m^3^)	169	18	1020	95	144	199	115
Meteorological measure (24-h average)							
Temperature (°C)	15.6	-5.0	33.9	6.5	16.6	24.2	9.9
Relative humidity (%)	61	16	97	49	61	74	17

Spearman correlation coefficients between air pollutants and meteorological factors were presented in Table 2. Except for humidity with PM_2.5_, CO and temperature, there were moderate positive correlation coefficients between pollutants and two meteorological factors.

**Table 2:** Spearman correlation coefficients between daily air pollutant concentrations and weather conditions.

	**PM**_**2.5**_	**SO**_**2**_	**NO**_**2**_	**O**_**3**_	**CO**	**Temperature**	**Humidity**
**PM**_**10**_	0.920**	0.719**	0.762**	-0.206**	0.752**	-0.469**	-0.199**
**PM**_**2.5**_		0.712**	0.723**	-0.226**	0.791**	-0.492**	0.024
**SO**_**2**_			0.769**	-0.414**	0.785**	-0.766**	-0.259**
**NO**_**2**_				-0.165**	-0.725**	-0.497**	-0.184**
**O**_**3**_					-0.454**	0.778**	-0.130**
**CO**						-0.730**	-0.056
**Temperature**							-0.001

**P<0.01

Table 3 summarizes the all-, sex- and age- specific effects for each pollutant on the concurrent days. Positive and statistically significant relationships were observed with both SO_2_ (*P*<0.0001) and NO_2_ (*P*<0.001), the corresponding increases were 3.55% (95%Cl: 1.93%, 5.18%) and 3.17% (95%Cl: 1.41%, 4.93%) with per 10μg/m^3^ increase in the concentrations of pollutants. Unexpectedly, O_3_ had negative association with epilepsy (*P*<0.05): a 10 μg/m^3^ increase in it was associated with a 0.84% (95%Cl: -1.58%, -0.09%) decrease in outpatient-visits. In sex-specific analysis, males showed stronger associations for SO_2_ (*P*<0.0001) and O_3_ (*P*<0.01) than females. However, only NO_2_ (*P*<0.005) showed more significant association in females. In age-specific analysis, the effects of NO_2_ and SO_2_ on children (aged <18) tended to be stronger than those on adult (aged 18–59) and elderly (aged>59) groups. In contrast, the effect tended to be strongest in adult group (aged 18–59) for O_3_. Interestingly, there was no significant effect of air pollutants on elderly group.

**Table 3:** Percent change (mean and 95% confidence interval) in daily outpatient-visits for epilepsy associated with a 10 μg/m^3^ (PM_10_, PM_2.5_, SO_2_, NO_2_, and O_3_) or 0.1 mg/m^3^ (CO) increase of air pollutants on the concurrent days in single-pollutant models.

		PM_10_	PM_2.5_	SO_2_	NO_2_	O_3_	CO
**All**		0.14 (-0.13, 0.41)	0.20 (-0.23, 0.62)	3.55 (1.93, 5.18)***	3.17 (1.41, 4.93)***	-0.84 (-1.58, -0.09)*	0.11 (-0.37, 0.59)
**Sex**	Male	0.22 (-0.08, 0.52)	0.27 (-0.21, 0.74)	3.81 (1.98, 5.64)***	3.13 (1.13, 5.12)**	-0.89 (-1.73, -0.04)*	0.16 (-0.38, 0.70)
	Female	0.01 (-0.33, 0.35)	0.09 (-0.44, 0.62)	3.17 (1.14,5.19)**	3.23 (1.05, 5.40)**	-0.77 (-1.68, 0.15)	0.03 (-0.57, 0.62)
**Age**	<18	0.13 (-0.22, 0.48)	0.31 (-0.24, 0.85)	4.64 (2.54, 6.74)***	4.55 (2.28, 6.82)***	-0.82 (-1.77, 0.14)	0.28 (-0.34, 0.90)
	18–59	0.21 (-0.09, 0.51)	0.23 (-0.26, 0.71)	2.95 (1.09, 4.81)**	2.87 (0.86, 4.88)**	-0.87 (-1.73, -0.02)*	0.03 (-0.51, 0.58)
	>59	-0.60 (-1.42, 0.22)	-1.01 (-2.29, 0.27)	2.54 (-2.04, 7.11)	-3.14 (-8.29, 2.01)	-0.58 (-2.72, 1.56)	-0.26 (-1.65, 1.13)

*P<0.05

**P<0.01

***P<0.001

**Fig 1:**
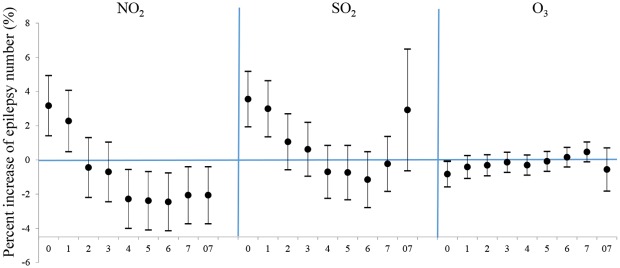
Percent increase in number of daily outpatient-visits for epilepsy associated with a 10-μg/m^3^ increase of air pollutants using different lag days in single-pollutant models. Values are reported as means and 95% confidence intervals. Abbreviations: Lag 07 day-moving average concentrations.

Fig 1 shows the results from the single-lag day (lag0-lag7) and cumulative exposure models (lag 07) for the percent increase of epilepsy number with per 10μg/m^3^ increase in pollutants. The most significant effects were evidenced on the concurrent days (lag0). The obvious effects of NO_2_, SO_2,_ and O_3_ concentration were limited on the concurrent and lag 1 days, with each 10μg/m^3^ increase of NO_2_, SO_2_ and O_3_ corresponded to 3.17% (95%Cl: 1.41%, 4.93%), 3.55% (95%Cl: 1.93%, 5.18%) and -0.84% (95%Cl: -1.58%, -0.09%) increase of outpatient-visits at lag 0 and 2.27% (95%Cl: 0.48%, 4.07%), 2.99% (95%Cl: 1.35%, 4.63%) increase at lag 1 for NO_2_ and SO_2_. In particular, negative association between epilepsy and NO_2_ was evidenced after lag 3, which might be a random and unexplainable result.

**Fig 2:**
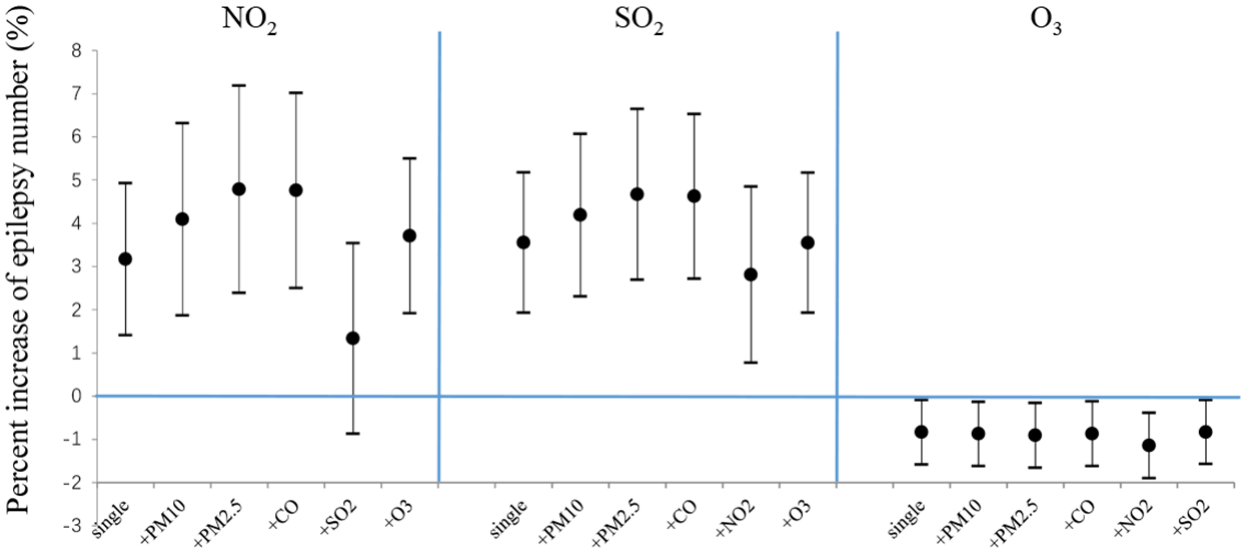
Percent increase in number of daily outpatient-visits for epilepsy associated with a 10-μg/m^3^ increase of air pollutants (NO_2_, SO_2_, and O_3_) in two-pollutant models. Values are reported as means and 95% confidence intervals.

Fig 2 shows the association between number of epilepsy patients and increase of NO_2_, SO_2_ and O_3_ concentrations in two-pollutant models. Except for the adjustment of NO_2_ for SO_2_, all pollutants’ associations were fairly stable after adjusted with others. The associations between epilepsy visits and NO_2_ as well as SO_2_ were more significant after adjustment for PM_2.5_ (4.79% (95%Cl: 2.39%, 7.18%), 4.19% (95Cl: 2.31%, 6.07%)). As for O_3_, the estimated decrease effect was at -1.14% (95%Cl: -1.90%, -0.39%) when adjusted for NO_2_.

**Fig 3:**
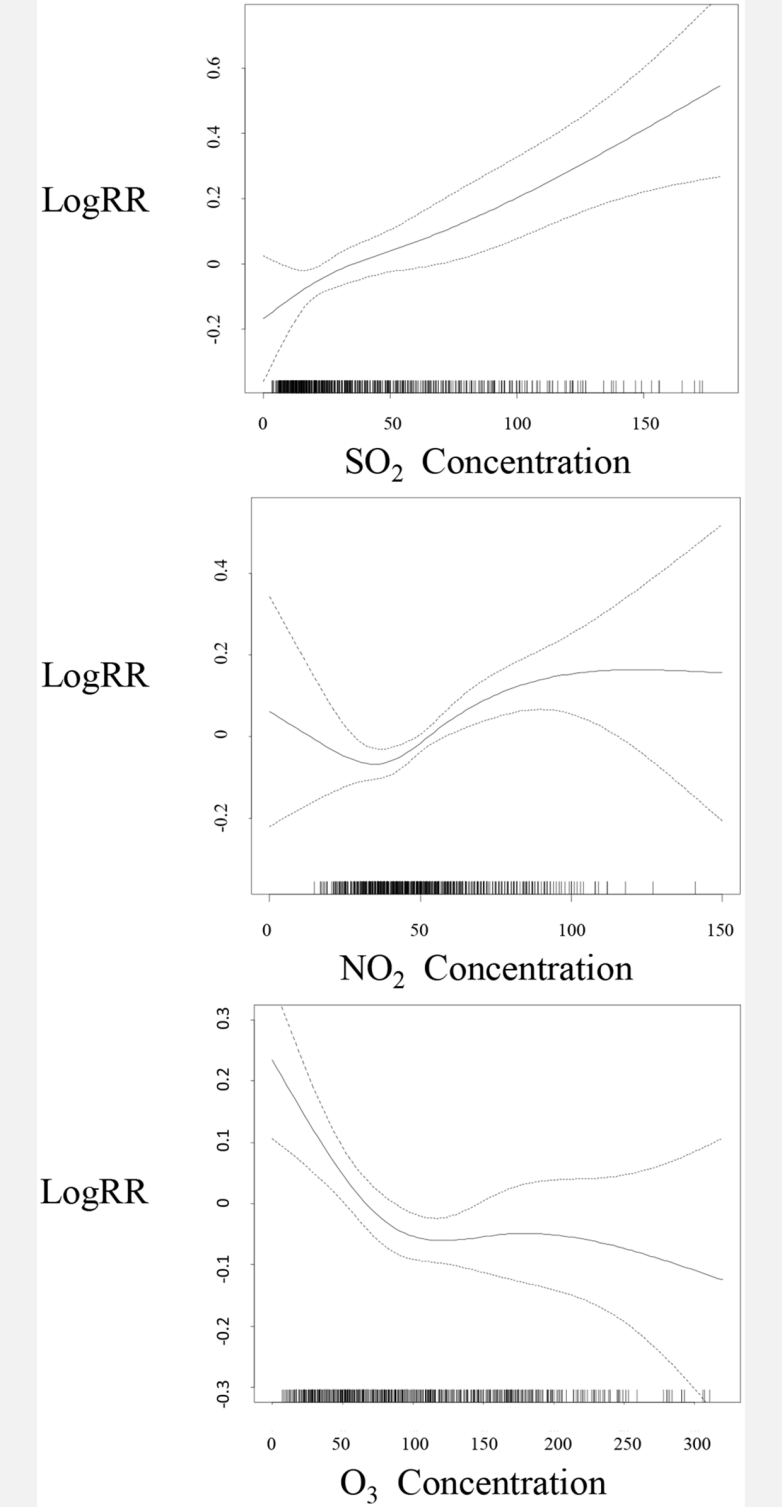
Smoothing plots of air pollution. X-axis is the concentration of pollutants (μg/m3). The estimated mean percentage of change in daily outpatient-visits for epilepsy is shown by the solid line, and the dotted lines represent twice the point-wise standard error.

Fig 3 shows that while the exposure–response relationships for NO_2_ and SO_2_ were generally linear and positive, O_3_ was generally linear and negative with outpatient-visits. From these curves, we did not observe any obvious threshold concentration below which there were no effects.

## Discussion

In the present study, SO_2_ and NO_2_ had positive relationships with epilepsy visits. Unexpectedly, there was negative relationship between O_3_ and epilepsy outpatient-visits. The associations were significant for both males and females, and were more significant for children (aged <18) and adults (aged 18–59) than for the elder (aged>59). When adjusted for PM_2.5_, the effects of NO_2_ and SO_2_ were more obvious. As for O_3_, we found stronger “protective” effect after adjustment for NO_2_. The lag model showed that the most significant effects were evidenced on concurrent days. Specially, we found a negative association between epilepsy and NO_2_ after lag 3, which might be the random and unexplainable result. Considering NO_2_ always produces acute health effects, we inclined to focus on the effects on the concurrent days while the results from lag models were used as references.

Before our study, only one study reported that epilepsy was influenced by air pollution. In Chile, Cakmak *et al* found that CO, O_3_, SO_2_, NO_2_, PM_10_, and PM2.5 were all positively associated with epilepsy hospitalization, and the results were not significantly influenced by age, sex, or season [12]. Different from that, only SO_2_ and NO_2_ were positively associated with epilepsy outpatient-visits here. More interestingly, we showed that O_3_ was negatively associated with epilepsy visits, suggesting that O_3_ might be a “protective” factor against epilepsy. The difference between our results and those of Cakmak *et al* might be due to the differences in races, climate, air pollution, or health care levels.

NO_2_ is an important part of automobile exhaust. Heretofore, the direct evidence of epilepsy induced by NO_2_ is limited. In the south of Spain, even low level NO_2_ exposure and traffic-related air pollution had side-effects on neurodevelopment, especially in children [13]. NO_2_ exposure also induces the changes of triglyceride, free fatty acids, esterified fatty acid, ganglioside, lipase activity and lipid peroxidation, and eventually leads to impaired nerve function in central nervous system [14,15]. Our present work added new evidence concerning the neurotoxic effects of NO_2_.

SO_2_ is a well-known irritant confirmed to induce respiratory responses. It has also been linked to nervous system disorders such as stroke and headache [16,17]. Cakmak *et al* have demonstrated the positive relationship between SO_2_ and epilepsy [12], which was consistent with our findings. The neurotoxic effect of SO_2_ has also been supported by animal studies. Sang *et al* reported the toxic effects of SO_2_ on hippocampus, including protein oxidation, DNA-protein crosslinks, and apoptosis [18]. Further work showed that SO_2_ may cause synaptic injuries in hippocampus via its derivatives [19]. More recently, chronic SO_2_ inhalation above environmental standard has been found to induce neurotoxicities via neuroinflammation [20]. In summary, SO_2_ exposure might be an environmental risk factor for epilepsy attacks. However, the mechanisms involved needed to be further clarified.

Though the stratospheric O_3_ layer protects human health against excess solar ultraviolet radiation [21], the O_3_ in earth's surface air can be dangerous. However, in the present study, negative association was observed between ambient O_3_ and epilepsy. This can be supported by a series of previous studies. León *et al* provided the first experimental evidence that repeated ozone administration at atoxic doses induces adaptation to oxidative stress, enabling animals to maintain hepatocellular integrity in response to CCl_4_ poisoning [22]. Controlled ozone administration also promotes oxidative preconditioning, preventing the damage induced by reactive oxygen species (ROS) and protecting against liver ischaemia-reperfusion (I/R) injury [23]. For epilepsy, the activated A1 adenosine receptors (A1Rs) play a pivotal role in antiepileptic and neuroprotective functions [24] while antioxidant/prooxidant imbalance is an important factor for epilepsy [25]. Ozone can exert its protective effects against pentylenetetrazole (PTZ)-induced epilepsy by re-establishment of cellular redox balance and A1Rs [25]. With the development of clinical applications, reasonability and effectiveness of O_3_ therapy are gradually being understood [26]. Medical O_3_, a gas mixture of O_3_ and O_2_, has been used as a curative agent for the treatment of different diseases over 150 years [27]. For mechanisms, O_3_ has therapeutic properties such as antimicrobial, anti-inflammatory, modulation of antioxidant defense system and apoptosis [27]. By using O_2_/O_3_ mixture to re-infusion of autologous blood slowly, ozonated autohemotherapy (OA) has favorable effects on nervous systems diseases, such as neurologic recovery in spontaneous spinal epidural hematoma [28] and antidepressant effect [29]. OA can also improve the level of brain-derived neurotrophic factor in patients with cognitive impairment [29]. Moreover, various cerebrovascular diseases are important causes in epilepsy, especially ischemic stroke [30], while ozone therapy has a potential role in the treatment of ischemic disorders [31,32]. However, the neurotoxic effects of excess O_3_ exposure are also well-known. Oxidative stress is involved in many neurodegenerative diseases, in which the over-production of O_3_ leads to both pathological and functional brain injuries [33]. Moreover, the environmental O_3_ exposure is associated with a series of adverse effects within the central nervous system, such as decreased cognitive response, decrease in motor activity and headaches, disturbances in the sleep-wake cycle, cell degeneration, and neurochemical alterations [34]. Taken together, the paradoxical effects of O_3_ may depend on its concentration: while high concentration of O_3_ may impair nervous system, low level of O_3_ may act as a protector. However, more studies are needed to further confirm that.

Nevertheless, limitations should also be mentioned. First, the study design is ecological in nature, which limits its ability for causal inference. The precise personal exposure level of air pollution may differ based on the location, time spent out side, and other factors. Just like most previous time-series studies, we used the average levels to represent the general exposure levels, which may not accurately reflect individual level associations. Though such ecological study design alone cannot demonstrate causality, it provides results worthy of further research [35]. Second, the data were only from urban areas of one city, which made it difficult to generalize the results to rural areas or to other cities. Third, more individual information such as BMI, smoking habits, drug history were unavailable, so we only performed stratified analyses by the data we could get, including sex and age. Fourth, our health data were collected based on two years of the data from only a single hospital, which may not represent the complete situation. Future work with data based on wider areas and longer periods may be helpful to further clarify the association between air pollution and epilepsy attacks. To establish the causal relationship between air pollutants and epilepsy, further epidemiological designs such as cohort studies can be performed in which personal exposure levels can be monitored with more accurate measures (e.g. personal monitors).

Conclusively, we demonstrated that short-term exposure to NO_2_ and SO_2_ were associated with increased outpatients for epilepsy. On the contrary, O_3_ was associated with decreased risk of epilepsy, suggesting that O_3_ might be a “protective” factor. Our data may contribute to the limited information concerning the effects of air pollution on epilepsy attacks.

## Supporting Information

S1 Fig Partial autocorrelation functions for the residuals from the time series analyses with 7 degrees of freedom per year. Dashed horizontal lines show the test that the apparent autocorrelation is non-zero, suggesting that the basic model may be appropriate.
(TIF)

S2 Fig Percent change in daily outpatient-visits for epilepsy associated with a 10 μg/m^3^ (PM_10_, PM_2.5_, SO_2_, NO_2_, and O_3_) or 0.1 mg/m^3^ (CO) increase of air pollutants using temperature lag model. The associations between pollutants and epilepsy were still existing, suggesting that the basic model was steady.
(TIF)

S1 Table The results of checking different degree of freedoms per year for time trends by Akaka’s Information Criterion (AIC). (DOC)

S2 Table Percent change (mean and 95% confidence interval) in daily outpatient-visits for epilepsy associated with a 10 μg/m^3^ (NO_2_, SO_2_, and O^3^) increase of air pollutants on the concurrent days with different degree of freedoms per year. (DOC)

## References

[1] Goodarzi P, Aghayan HR, Soleimani M, Norouzi-Javidan A, Mohamadi-Jahani F, Jahangiri S, et al Stem cell therapy for treatment of epilepsy. Acta Med Iran. 2014; 52: 651–655. 25325201

[2] Ngugi AK, Bottomley C, Kleinschmidt I, Sander JW, Newton CR. Estimation of the burden of active and life-time epilepsy: a meta-analytic approach. Epilepsia. 2010; 51: 883–890. 10.1111/j.1528-1167.2009.02481.x 20067507 PMC3410521

[3] Ye X, Peng L, Kan H, Wang W, Geng F, Mu Z, et al Acute Effects of Particulate Air Pollution on the Incidence of Coronary Heart Disease in Shanghai, China. PLoS One. 2016; 11: e0151119 10.1371/journal.pone.0151119 26942767 PMC4778855

[4] Xu Q, Li X, Wang S, Wang C, Huang F, Gao Q, et al Fine Particulate Air Pollution and Hospital Emergency Room Visits for Respiratory Disease in Urban Areas in Beijing, China, in 2013. PLoS One. 2016; 11: e0153099 10.1371/journal.pone.0153099 27054582 PMC4824441

[5] Calderon-Garciduenas L, Kulesza RJ, Doty RL, D'Angiulli A, Torres-Jardon R. Megacities air pollution problems: Mexico City Metropolitan Area critical issues on the central nervous system pediatric impact. Environ Res. 2015; 137: 157–169. 10.1016/j.envres.2014.12.012 25543546

[6] Migliore L, Coppede F. Environmental-induced oxidative stress in neurodegenerative disorders and aging. Mutat Res. 2009; 674: 73–84. 10.1016/j.mrgentox.2008.09.013 18952194

[7] Kan H, Chen B, Hong C. Health impact of outdoor air pollution in China: current knowledge and future research needs. Environ Health Perspect. 2009; 117: A187 10.1289/ehp.12737 19478975 PMC2685855

[8] Chen B, Kan H, Chen R, Jiang S, Hong C. Air pollution and health studies in China—policy implications. J Air Waste Manag Assoc. 2011; 61: 1292–1299. 22168112 10.1080/10473289.2011.604288

[9] Cao J, Xu H, Xu Q, Chen B, Kan H. Fine particulate matter constituents and cardiopulmonary mortality in a heavily polluted Chinese city. Environ Health Perspect. 2012; 120: 373–378. 10.1289/ehp.1103671 22389181 PMC3295342

[10] Lu F, Zhou L, Xu Y, Zheng T, Guo Y, Wellenius GA, et al Short-term effects of air pollution on daily mortality and years of life lost in Nanjing, China. Sci Total Environ. 2015; 536: 123–129. 10.1016/j.scitotenv.2015.07.048 26204048

[11] Cai J, Zhao A, Zhao J, Chen R, Wang W, Ha S, et al Acute effects of air pollution on asthma hospitalization in Shanghai, China. Environ Pollut. 2014; 191: 139–144. 10.1016/j.envpol.2014.04.028 24836410

[12] Cakmak S, Dales RE, Vidal CB. Air pollution and hospitalization for epilepsy in Chile. Environ Int. 2010; 36: 501–505. 10.1016/j.envint.2010.03.008 20452673

[13] Freire C, Ramos R, Puertas R, Lopez-Espinosa MJ, Julvez J, Aguilera I, et al Association of traffic-related air pollution with cognitive development in children. J Epidemiol Community Health. 2010; 64: 223–228. 10.1136/jech.2008.084574 19679705

[14] Farahani H, Hasan M. Nitrogen dioxide induced changes in level of free fatty acids, triglyceride, esterified fatty acid, ganglioside and lipase activity in the guinea pig brain. J Environ Sci Health B. 1992; 27: 53–71. 1556389 10.1080/03601239209372767

[15] Farahani H, Hasan M. Effect of NO2 on lipids and lipid peroxidation in the CNS of the guinea-pig. Pharmacol Toxicol. 1990; 66: 146–149. 2315266 10.1111/j.1600-0773.1990.tb00722.x

[16] Szyszkowicz M, Rowe BH, Kaplan GG. Ambient sulphur dioxide exposure and emergency department visits for migraine in Vancouver, Canada. Int J Occup Med Environ Health. 2009; 22: 7–12. 10.2478/v10001-009-0006-7 19329386

[17] Shah AS, Lee KK, McAllister DA, Hunter A, Nair H, Whiteley W, et al Short term exposure to air pollution and stroke: systematic review and meta-analysis. BMJ. 2015; 350: h1295 10.1136/bmj.h1295 25810496 PMC4373601

[18] Sang N, Hou L, Yun Y, Li G. SO(2) inhalation induces protein oxidation, DNA-protein crosslinks and apoptosis in rat hippocampus. Ecotoxicol Environ Saf. 2009; 72: 879–884. 10.1016/j.ecoenv.2008.07.007 18722661

[19] Yun Y, Yao G, Yue H, Guo L, Qin G, Li G, et al SO(2) inhalation causes synaptic injury in rat hippocampus via its derivatives in vivo. Chemosphere. 2013; 93: 2426–2432. 10.1016/j.chemosphere.2013.08.063 24099899

[20] Yao G, Yue H, Yun Y, Sang N. Chronic SO2 inhalation above environmental standard impairs neuronal behavior and represses glutamate receptor gene expression and memory-related kinase activation via neuroinflammation in rats. Environ Res. 2015; 137: 85–93. 10.1016/j.envres.2014.11.012 25498917

[21] Diffey B. Climate change, ozone depletion and the impact on ultraviolet exposure of human skin. Phys Med Biol. 2004; 49: R1–11. 14971768 10.1088/0031-9155/49/1/r01

[22] Leon OS, Menendez S, Merino N, Castillo R, Sam S, Perez L, et al Ozone oxidative preconditioning: a protection against cellular damage by free radicals. Mediators Inflamm. 1998; 7: 289–294. 9792340 10.1080/09629359890983 PMC1781855

[23] Ajamieh H, Merino N, Candelario-Jalil E, Menendez S, Martinez-Sanchez G, Re L, et al Similar protective effect of ischaemic and ozone oxidative preconditionings in liver ischaemia/reperfusion injury. Pharmacol Res. 2002; 45: 333–339. 12030798 10.1006/phrs.2002.0952

[24] Swiader MJ, Kotowski J, Luszczki JJ. Modulation of adenosinergic system and its application for the treatment of epilepsy. Pharmacol Rep. 2014; 66: 335–342. 10.1016/j.pharep.2013.10.005 24905507

[25] Mallok A, Vaillant JD, Soto MT, Viebahn-Hansler R, Viart Mde L, Perez AF, et al Ozone protective effects against PTZ-induced generalized seizures are mediated by reestablishment of cellular redox balance and A1 adenosine receptors. Neurol Res. 2015; 37: 204–210. 10.1179/1743132814Y.0000000445 25258110

[26] Bocci VA. Scientific and medical aspects of ozone therapy. State of the art. Arch Med Res. 2006; 37: 425–435. 16624639 10.1016/j.arcmed.2005.08.006

[27] Sancak EB, Turkon H, Cukur S, Erimsah S, Akbas A, Gulpinar MT, et al Major Ozonated Autohemotherapy Preconditioning Ameliorates Kidney Ischemia-Reperfusion Injury. Inflammation. 2016; 39: 209–217. 10.1007/s10753-015-0240-z 26282390

[28] Yu L, Lu X, Shi H, Wang Q. Does ozone autohemotherapy have positive effect on neurologic recovery in spontaneous spinal epidural hematoma? Am J Emerg Med. 2014; 32: 949 e941–942. 10.1016/j.ajem.2014.01.039 24602896

[29] Coppola L, Luongo C, Pastore A, Masciello C, Parascandola RR, Mastrolorenzo L, et al Ozonized autohaemotransfusion could be a potential rapid-acting antidepressant medication in elderly patients. Int J Geriatr Psychiatry. 2010; 25: 208–213. 10.1002/gps.2322 19521954

[30] Berg AT. Seizures and Epilepsy after Ischemic Stroke. Epilepsy Curr. 2003; 3: 129–130. 15309055 10.1046/j.1535-7597.2003.03408.x PMC321199

[31] Clavo B, Catala L, Perez JL, Rodriguez V, Robaina F. Ozone Therapy on Cerebral Blood Flow: A Preliminary Report. Evid Based Complement Alternat Med. 2004; 1: 315–319. 15841265 10.1093/ecam/neh039 PMC538510

[32] Clavo B, Suarez G, Aguilar Y, Gutierrez D, Ponce P, Cubero A, et al Brain ischemia and hypometabolism treated by ozone therapy. Forsch Komplementmed. 2011; 18: 283–287. 10.1159/000333795 22105041

[33] Rivas-Arancibia S, Guevara-Guzman R, Lopez-Vidal Y, Rodriguez-Martinez E, Zanardo-Gomes M, Angoa-Perez M, et al Oxidative stress caused by ozone exposure induces loss of brain repair in the hippocampus of adult rats. Toxicol Sci. 2010; 113: 187–197. 10.1093/toxsci/kfp252 19833740

[34] Martinez-Lazcano JC, Gonzalez-Guevara E, del Carmen Rubio M, Franco-Perez J, Custodio V, Hernandez-Ceron M, et al The effects of ozone exposure and associated injury mechanisms on the central nervous system. Rev Neurosci. 2013; 24: 337–352. 10.1515/revneuro-2012-0084 23585211

[35] Morgenstern H. Ecologic studies in epidemiology: concepts, principles, and methods. Annu Rev Public Health. 1995; 16: 61–81. 7639884 10.1146/annurev.pu.16.050195.000425

