# Correction: The Novel Relationship between Urban Air Pollution and Epilepsy: A Time Series Study

**DOI:** 10.1371/journal.pone.0165902

**Published:** 2016-10-27

**Authors:** Chen Xu, Yan-Ni Fan, Hai-Dong Kan, Ren-Jie Chen, Jiang-Hong Liu, Ya-Fei Li, Yao Zhang, Ai-Ling Ji, Tong-Jian Cai

The following information is missing from the Funding section: This article was supported by National Natural Science Foundation of China (81372952, 81202220, 81472190). The funder had no role in study design, data collection and analysis, decision to publish, or preparation of the manuscript.

## References

[1] Xu C, Fan Y-N, Kan H-D, Chen R-J, Liu J-H, Li Y-F, et al (2016) The Novel Relationship between Urban Air Pollution and Epilepsy: A Time Series Study. PLoS ONE 11(8): e0161992 doi:10.1371/journal.pone.0161992 27571507 10.1371/journal.pone.0161992 PMC5003346

