# Supplementary figures and images for: The Novel Relationship between Urban Air Pollution and Epilepsy: A Time Series Study
Source: PLoS One. 2016 Aug 29;11(8):e0161992. doi: 10.1371/journal.pone.0161992 (PMC5003346; doi:10.1371/journal.pone.0161992)

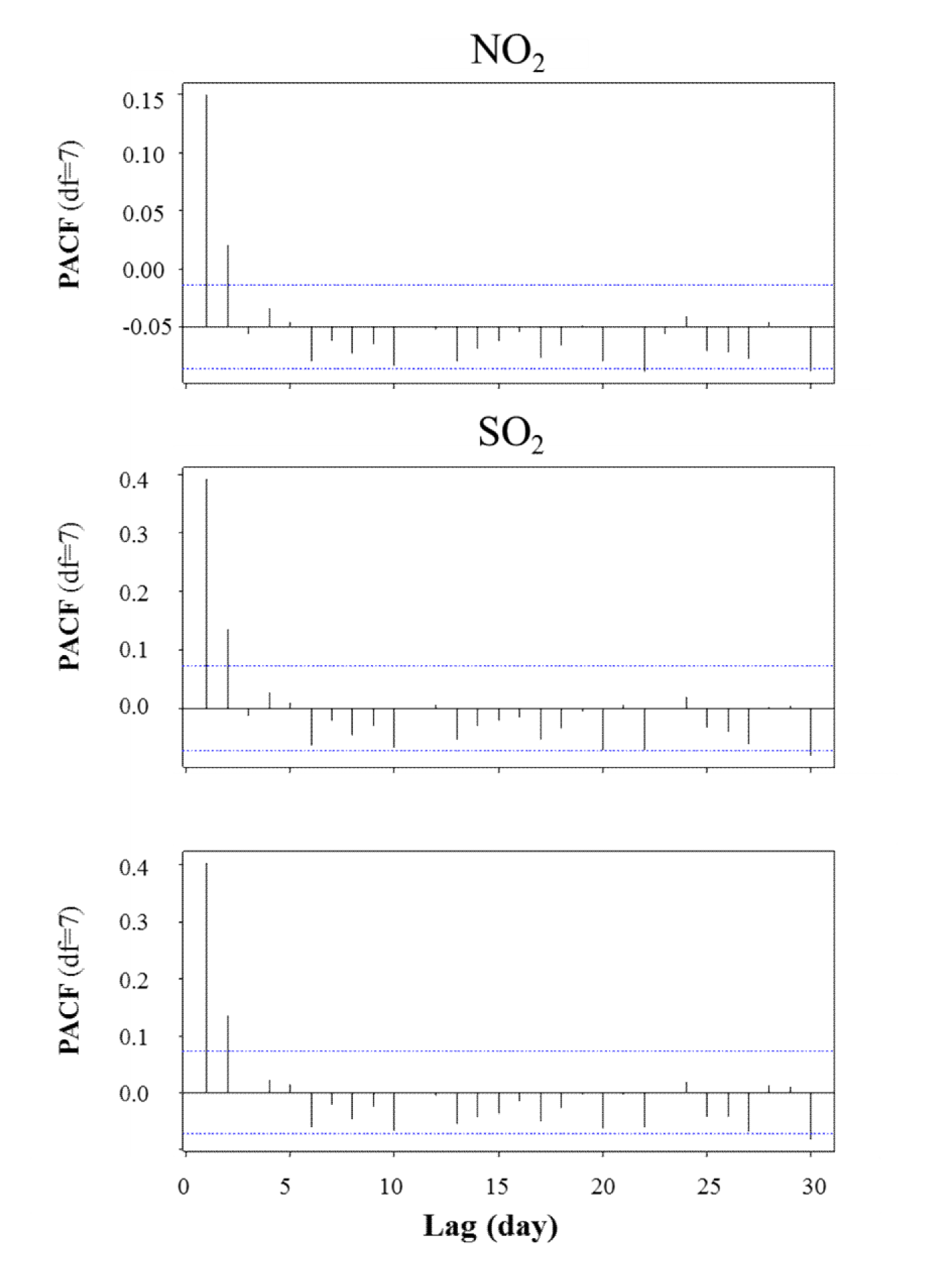

Supplement: S1 Fig — Dashed horizontal lines show the test that the apparent autocorrelation is non-zero, suggesting that the basic model may be appropriate. (TIF) [file pone.0161992.s001.tif]

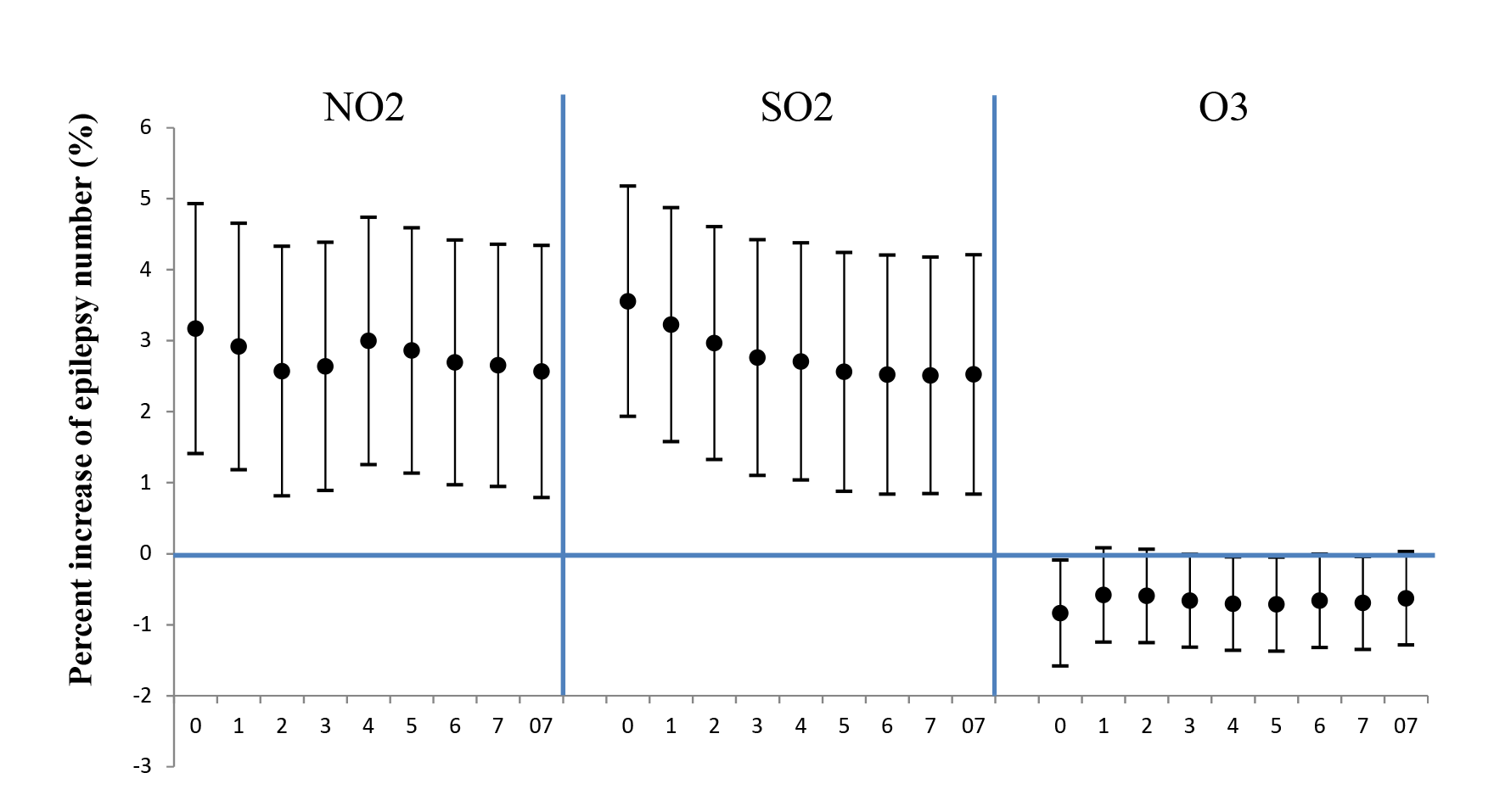

Supplement: S2 Fig — The associations between pollutants and epilepsy were still existing, suggesting that the basic model was steady. (TIF) [file pone.0161992.s002.tif]
