# Supplementary material for: The Novel Relationship between Urban Air Pollution and Epilepsy: A Time Series Study
Source: PLoS One. 2016 Aug 29;11(8):e0161992. doi: 10.1371/journal.pone.0161992 (PMC5003346; doi:10.1371/journal.pone.0161992)
Supplement: S1 Table — (DOC) [file pone.0161992.s003.doc]

**S1 Table.** The results of checking different degree of freedoms per year for time trends by Akaka’s Information Criterion (AIC).

| **df** | **SO_2_** | **NO_2_** | **O_3_** |
| --- | --- | --- | --- |
| 4 | 5935.918 | 5944.850 | 5962.331 |
| 5 | 5917.253 | 5926.873 | 5942.727 |
| 6 | 5912.552 | 5921.959 | 5941.622 |
| 7 | 5864.349 | 5859.848 | 5881.911 |
| 8 | 5844.604 | 5866.133 | 5884.431 |
| 9 | 5864.825 | 5876.869 | 5890.665 |
| 10 | 5865.239 | 5879.196 | 5894.521 |

Abbreviations: DF: degree of freedom
