# Supplementary material for: The Novel Relationship between Urban Air Pollution and Epilepsy: A Time Series Study
Source: PLoS One. 2016 Aug 29;11(8):e0161992. doi: 10.1371/journal.pone.0161992 (PMC5003346; doi:10.1371/journal.pone.0161992)
Supplement: S2 Table — (DOC) [file pone.0161992.s004.doc]

**S2 Table.** Percent change (mean and 95% confidence interval) in daily outpatient-visits for epilepsy associated with a 10 μg/m^3^ (NO_2_, SO_2_, and O^3^) increase of air pollutants on the concurrent days with different degree of freedoms per year.

| **df** | **NO_2_** | **SO_2_** | **O_3_** |
| --- | --- | --- | --- |
| 4 | 2.91 (1.16, 4.66)*** | 3.05 (1.45, 4.64)*** | -0.77 (-1.52, -0.03)* |
| 5 | 2.90 (1.11, 4.61)*** | 3.08 (1.46, 4.70)*** | -0.78 (-1.53, -0.04)* |
| 6 | 3.06 (1.31, 4.81)*** | 3.24 (1.62, 4.87)*** | -0.79 (-1.54, -0.05)* |
| 7 | 3.17 (1.41, 4.93)*** | 3.55 (1.93, 5.18)*** | -0.84 (-1.58, -0.09)* |
| 8 | 3.18 (1.39, 4.97)*** | 3.50 (1.88, 5.13)*** | -0.80 (-1.54, -0.04)* |
| 9 | 3.21 (1.40, 5.02)*** | 3.53 (1.91, 5.16)*** | -0.82 (-1.58, -0.06)* |

**P<*0.05

****P<*0.001

Abbreviations: DF: degree of freedom
